# Function and evolution of channels and transporters in photosynthetic membranes

**DOI:** 10.1007/s00018-013-1412-3

**Published:** 2013-07-09

**Authors:** Bernard E. Pfeil, Benoît Schoefs, Cornelia Spetea

**Affiliations:** 1 Department of Biological and Environmental Sciences, University of Gothenburg, 40530 Gothenburg, Sweden; 2 Mer, Molécules, Santé, MicroMar - EA2160, LUNAM Université, IUML – FR 3473 CNRS, University of Le Mans, 72085 Le Mans Cedex 9, France

**Keywords:** Channel, Chloroplast, Cyanobacteria, Phylogeny, Thylakoid membrane, Transporter

## Abstract

Chloroplasts from land plants and algae originated from an endosymbiotic event, most likely involving an ancestral photoautotrophic prokaryote related to cyanobacteria. Both chloroplasts and cyanobacteria have thylakoid membranes, harboring pigment-protein complexes that perform the light-dependent reactions of oxygenic photosynthesis. The composition, function and regulation of these complexes have thus far been the major topics in thylakoid membrane research. For many decades, we have also accumulated biochemical and electrophysiological evidence for the existence of solute transthylakoid transport activities that affect photosynthesis. However, research dedicated to molecular identification of the responsible proteins has only recently emerged with the explosion of genomic information. Here we review the current knowledge about channels and transporters from the thylakoid membrane of *Arabidopsis thaliana* and of the cyanobacterium *Synechocystis* sp. PCC 6803. No homologues of these proteins have been characterized in algae, although similar sequences could be recognized in many of the available sequenced genomes. Based on phylogenetic analyses, we hypothesize a host origin for most of the so far identified *Arabidopsis* thylakoid channels and transporters. Additionally, the shift from a non-thylakoid to a thylakoid location appears to have occurred at different times for different transport proteins. We propose that closer control of and provision for the thylakoid by products of the host genome has been an ongoing process, rather than a one-step event. Some of the proteins recruited to serve in the thylakoid may have been the result of the increased specialization of its pigment-protein composition and organization in green plants.

**Electronic supplementary material:**

The online version of this article (doi:10.1007/s00018-013-1412-3) contains supplementary material, which is available to authorized users.

## Introduction

**Fig. 1:**
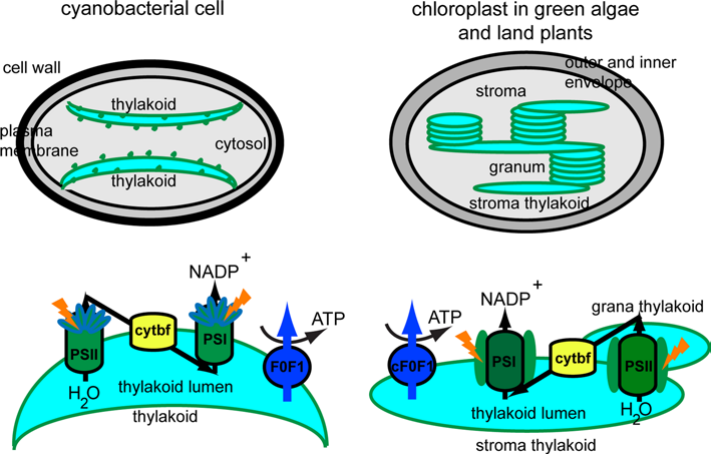
Thylakoid membrane organization and composition in cyanobacteria and plants. Cyanobacterial thylakoid membranes are located directly in the cytosol, are arranged in layers, make contact with the plasma membrane, and have attached phycobilisomes. Thylakoid membranes in green algae and land plants are located inside the chloroplast, are organized in grana stacks interconnected by stroma-exposed lamellae and contain chlorophyll-protein complexes for harvesting light. Four photosynthetic complexes are present in the thylakoid membrane, namely photosystem II (PSII), cytochrome b_6_f (cytbf), photosystem I (PSI) and H^+^-translocating ATP synthase (F_0_F_1_, cF_0_F_1_). These complexes are uniformly distributed in cyanobacteria, whereas in green algae and land plants they display a lateral distribution

Oxygenic photosynthesis is a biophysicochemical process that converts carbon dioxide into organic compounds using light as a source of energy. It occurs in plants, algae and cyanobacteria, but not in archaea, and uses water as a source of electrons, releasing oxygen as a waste product. Photosynthesis confers autotrophy to organisms and is the only natural process allowing for creation of food from simple and abundant compounds. This process is vital for all aerobic life on Earth because, in addition to maintaining normal levels of oxygen in the atmosphere, photosynthetic products directly or indirectly constitute the ultimate source of energy in food. Regardless of the type of photosynthetic organism, this process takes place according to the same scheme [1]. It always requires the collection of photons by pigment molecules within light-harvesting complexes. The harvested energy is transferred to the reaction center and is used to drive an electron flow within the membrane hosting the photosynthetic apparatus, the so-called thylakoid membrane. In prokaryotic photosynthetic organisms, such as cyanobacteria, thylakoid membranes are in direct contact with the cytosol, whereas in eukaryotic photosynthetic organisms, i.e., red algae, brown algae, diatoms, green algae and land plants, thylakoids are separated from the cytosol by envelopes, together forming a unique cell compartment, the chloroplast. Glaucophytes occupy an intermediate position between cyanobacteria and chloroplasts, because their photosynthetic apparatus, the so-called cyanelle, is surrounded by a peptidoglycan layer that is believed to be a relic of the endosymbiotic origin of chloroplasts from cyanobacteria. Thus, the chloroplast has evolved via primary symbiosis from an ancestor shared with cyanobacteria. As a result of secondary symbiosis, a four-layer envelope surrounds the chloroplast in brown algae and diatoms, in contrast to a double envelope around the chloroplast in red algae, green algae and land plants [2, 3]. The organization of the photosynthetic machinery in cyanobacteria, green algae and land plants is presented in Fig. 1.

The photosynthetic apparatus is composed of four macrocomplexes, namely the water-oxidizing photosystem II (PSII), cytochrome b_6_f, photosystem I (PSI), and the H^+^-translocating ATP synthase (CF_0_F_1_) [4]. They supply ATP and NADPH for the synthesis of many essential compounds, such as carbohydrates, for autotrophic growth. A photosystem is a pigment-protein complex composed of a reaction center (RC) and a light-harvesting complex (LHC). Two families of pigments are found in LHCs, namely tetrapyrroles and carotenoids. The presence of open-chain tetrapyrroles (phycobilins) is restricted to the phycobilisomes in cyanobacteria, glaucophytes and red algae, whereas closed tetrapyrroles (chlorophylls) are found in all photosynthetic organisms (Table 1). The phycobilisome is the major LHC in cyanobacteria, glaucophytes and red algae, and is composed of several pigment-protein complexes linked together by colorless proteins [5]. The main phycobilins of cyanobacteria and red algae are phycocyanin, phycoerythrobilin and allophycocyanin. The most ubiquitous chlorophyll type is Chl *a,* a mandatory pigment in both RC and LHC [6]. In all photosynthetic organisms, except for most cyanobacteria and red algae, Chl *a* is aided in its task of harvesting light by accessory pigments, namely other types of Chl and carotenoids (Table 1). The purpose of accessory pigments is to enlarge the range of wavelengths collected by LHCs [6]. In addition to their role in harvesting light, carotenoids play crucial roles in thylakoid organization [7], in photoprotection of Chl molecules and dissipation of excess energy, for instance, through operation of the xanthophyll cycle [8–10]. Despite the distinct carotenoid composition of brown algae, diatoms, green algae and land plants (Table 1), they all share a role in photoprotection that includes the xanthophyll cycle [10]. Due to lack of space, a detailed comparison of the arrangement of pigments within PSI, PSII and associated LHCs will not be described here, but the interested reader will find relevant information in several recent reviews [6, 11–13]. For the scope of this introduction, it is sufficient to know that the RCII and RCI structures and functions are essentially conserved across photosynthetic organisms. If both types of RC are membrane-embedded, LHCs can be either exposed at the outer surface of the thylakoid membrane, as in cyanobacteria and red algae, or embedded in the thylakoid membrane (Fig. 1).

**Table 1:** Main chlorophyll and carotenoid types in the various taxons of photosynthetic organisms

Pigment type	Cyanobacteria	Glaucophytes	Red algae	Brown algae	Diatoms	Green algae	Land plants
Phycobilisomes	+	+	+	−	−	−	−
Chl *a*	+ Except in *Acaryochloris marina* and related taxa	+	+	+	+	+	+
Chl *b*	+ Except in Prochlorophytes	−	−	−	−	+	+
Chl *c*	−	−	−	+	+	−	−
Chl *d*	Only in *A. marina* and related taxa	−	−	−	−	−	−
Chl *f*	Only in filamentous cyanobacteria from stromatolites	−	−	−	−	−	−
β-Carotene	+	+	Unicellular	+	+	+	+
Fucoxanthin	−	−	−	+	+	−	−
Diadinoxanthin	−	−	−	Traces	+	−	−
Diatoxanthin	−	−	−	Traces	+	−	−
Violaxanthin	−	−	+	+	+	+	+
Lutein	−		Macrophytes	−	−	+	+
Zeaxanthin	Depends on species	+	+	+	Traces	+	+
Echinenone	Depends on species	−	−	−	−	−	−
Myxoxanthophyll	Depends on species	−	−	−	−	−	−
Canthaxanthin	*Anabaena*	−	−	−	−	−	−
Xanthophyll cycle	−	−	−	+	+	+	+

The photosynthetic pigment composition has had a great impact on the evolution of thylakoid membrane organization, as reviewed below. Since chloroplasts have evolved from an ancestor shared with cyanobacteria, it was thought in the past that cyanobacterial thylakoid membranes are completely separated from the plasma membrane, as is the case with chloroplast thylakoids and the inner envelope. However, various internal membrane organizations have been found in cyanobacteria. In most cases, three to eight thylakoid membrane pairs merge together at a site close to the plasma membrane (Fig. 1; [14]). Regardless of the organization of thylakoids, the presence of large phycobilisomes, which protrude from the thylakoid lamellae towards the cytoplasm, prevents membrane stacking [15]. A similar arrangement of thylakoids was found in red algae [16, 17]. In diatoms and brown algae, chloroplast thylakoid membranes are loosely appressed and organized into groups of three [17, 18]. However, in the chloroplast of green algae and land plants, cylindrical stacks of 5–20 thylakoids (grana) are interconnected by non-appressed, so-called stroma thylakoid membranes (Fig. 1; [19, 20]). According to the 3D-organization of the thylakoid membrane based on electron tomography [21, 22], grana should be seen as stacks of straight, exactly parallel pairs of thylakoid membranes, each pair of grana disks merging with stroma lamellae by staggered membrane protrusions.

For a long time it was thought that the different macrocomplexes comprising the photosynthetic apparatus were organized linearly along thylakoid membranes. This view is no longer valid since it has been established that the complexes may be located exclusively in the grana (active PSII), exclusively in the stroma-exposed thylakoids (PSI, inactive PSII, ATP synthase) or in both types of membranes (cytochrome b_6_f complex) [23]. This organization is restricted to green algae and land plants, since other algae, diatoms and cyanobacteria display a more uniform distribution of photosynthetic complexes (Fig. 1; [3]). The differences among photosynthetic organisms in thylakoid pigment-protein composition and membrane organization have implications for the optimal function, photoprotection and repair of photosynthetic complexes. Particularly, the intensively studied repair of PSII during light stress in plants and cyanobacteria has been shown to share common components involved in proteolytic degradation and de novo synthesis. Important differences have, however, also been reported, such as plant-specific phosphorylation of several PSII proteins and migration of damaged complexes between the grana and the stroma thylakoid regions, which is accompanied by changes in the oligomeric structure of those complexes [24]. Although less studied, the mechanism of PSII repair in green algae is thought to closely resemble the mechanism described for land plants [25]. In brown algae and diatoms, the mechanisms of PSII photoprotection and repair have only recently begun to be revealed [26, 27].

## The photosynthetic machinery requires thylakoid channels and transporters for correct functioning

Apart from photosynthetic macrocomplexes, thylakoid membranes harbor protein import complexes, auxiliary enzymes, channels and transporters. They are essential for biogenesis, optimal function, maintenance and repair of the photosynthetic machinery. Learning about the function(s) of these proteins can be critical for understanding the photosynthetic process itself and its regulation. In the cyanobacterial cell, channels and transporters mediate the exchange of solutes between the cytosol and the thylakoid lumen, whereas in the chloroplast of plants and algae, the exchange takes place between the stroma and the thylakoid lumen. All chloroplast thylakoid channels and transporters are nuclear-encoded proteins and, therefore, have to be imported into the chloroplast, and then inserted into the thylakoid membrane. In general, they carry a chloroplast-targeting sequence and share the structural features of membrane transporters, namely a hydrophobic structure, a pore-forming sequence, and molecule-binding sites. Using bioinformatics tools, these specific features can be used to predict homologues of membrane transporters from other organisms, as targeted to the chloroplast. Following sequencing of the *Arabidopsis* *thaliana* (L.) Heynh *(Arabidopsis)* genome [28], bioinformatic analyses have estimated that 4.5 % of its nuclear-encoded proteins represent membrane channels and transporters [29, 30].

According to the Transport Classification system (TC) available at the TCDB database [31] (http://www.tcdb.org/), transport proteins found in biological membranes belong to three major categories: channels/porins (TC #1), secondary transporters (TC #2), and primary transporters/pumps (TC #3). Apart from TCDB, there are several other excellent databases of membrane transporters, including PlantsT for functional genomics of plant transporters ([32] (http://plantst.genomics.purdue.edu/) and ARAMEMNON for sequence analysis of plant membrane proteins [33] (http://aramemnon.botanik.uni-koeln.de/).

In this review, we provide an update on channels and transporters from the thylakoid membrane of the cyanobacterium *Synechocystis* sp. PCC 6803 (*Synechocystis*) and *Arabidopsis*, that belong to the three major TC categories. Table 2 presents a summary of the current knowledge about the biochemical activity and physiological role of these types of transporters. We also perform a comprehensive phylogenetic analysis to reveal their origins and evolutionary history. Mass-spectrometry-based proteomics has been a very successful approach in identifying a tremendous number of chloroplast envelope channels and transporters [34, 35]. However, the identification of such proteins using the same approach in the thylakoid membrane has been less successful, hampered by the fact that they may represent only 0.5 % of the thylakoid proteome [36]. Furthermore, the detection of thylakoid transporters can be complicated by their likely restriction to the stroma-exposed thylakoid membranes, which represent about 10 % of the thylakoid membrane. Only four of the 11 channels and transporters in Table 2 have been identified by proteomics of thylakoid membranes, namely *Arabidopsis* TIP2;1, TAAC and KEA3 [34, 35] and *Synechocystis* NhaS3 [37]. Sequence-based prediction combined with experimental validation of the location of putative candidates has proven successful for the identification of most thylakoid transporters [38].

**Table 2:** Thylakoid channels and transporters from *Arabidopsis thaliana* and *Synechocystis* sp. PCC 6803

Protein name	Organism of origin	Accession number	Substrate specificity	Physiological role	References
**TC #1. Channels/porins**					
K^+^ channel (SynK)	*Synechocystis*	*slr0498*	K^+^	Photosynthesis: regulation of the electric component of proton motive force	[46, 51]
Two-pore K^+^ channel 3 (TPK3)	*Arabidopsis*	*At4g18160*	K^+^?	Not determined	[46, 47, 128]
Chloride channel e (CLCe)	*Arabidopsis*	*At4g35440*	Cl^−^, NO_2_^−^	Photosynthesis, nitrate homeostasis	[57, 58]
Tonoplast intrinsic protein 2;1 (TIP2;1)	*Arabidopsis*	*At3g16240*	H_2_O, H_2_O_2_, NH_3_, urea	Not determined	[34, 35, 61]
**TC #2. Secondary transporters**					
Phosphate transporter 4;1 (PHT4;1)	*Arabidopsis*	*At2g29650*	Pi	PSII repair and photoprotection	[66, 67, 71]
Thylakoid ATP/ADP carrier (TAAC)	*Arabidopsis*	*At5g01500*	ATP, ADP, PAPS, PAP	Thylakoid biogenesis, PSII repair and photoprotection, sulfur metabolism, retrograde signaling	[78, 79, 82, 84]
Na^+^/H^+^ antiporter NhaS3	*Synechocystis*	*sll0689*	Na^+^, H^+^	Essential gene. Balance of Na^+^/K^+^ ratio. Reduce toxic effects of Na^+^ in the cytosol and of lumen acidification	[86]
K^+^ exchange antiporter 3 (KEA3)	*Arabidopsis*	*At4g04850*	Not determined	Not determined	[35]
**TC #3. Primary transporters**					
CF_0_ ATPase AtpF Atp-G Atp-H Atp-I	*Arabidopsis*	*AtCg00130* *At4g32260* *AtCg00140* *AtCg00150*	H^+^	ATP supply for CO_2_ fixation and other energy-dependent chloroplast processes	[4]
Cu^2+^-transporting ATPase (PAA2)	*Arabidopsis*	*At5g21930*	Cu^2+^	Copper supply to the thylakoid lumen	[104]
Cu^2+^-transporting ATPase (PacS)	*Synechocystis*	*sll1920*		Copper supply to the thylakoid lumen	[105]

The classification according to the Transport Classification database (www.tcdb.org), gene accession numbers (according to www.arabidopsis.org and http://genome.kazusa.or.jp/cyanobase/Synechocystis), experimentally determined substrate and proposed physiological role are indicated. The source for the experimental data describing each thylakoid transport protein is also provided

### Channels

**Fig. 2:**
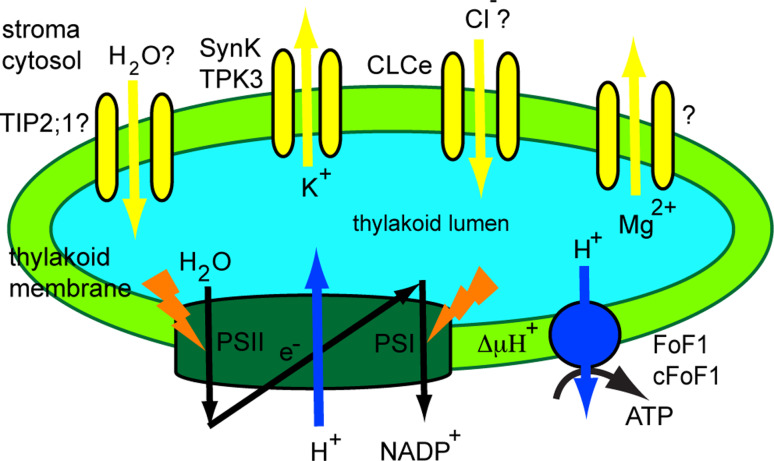
Function of thylakoid channels. A schematic representation of the photosynthetic electron transport from water to NADP^+^ is provided. Coupled H^+^ transport into the thylakoid lumen creates a H^+^ electrochemical gradient (ΔμH^+^). This is used by the H^+^-ATP synthase to generate ATP from ADP and Pi. Through their ion transport activity, the K^+^ and Cl^−^ channel maintain ΔμH^+^. The activity of a Mg^2+^ channel has also been demonstrated, but the responsible protein is unknown. A water channel belonging to the tonoplast intrinsic protein family TIP (TIP2;1) may also be located in the thylakoid and play a role in osmoregulation

Channels (TC #1) transport solutes down their concentration gradient without consuming energy and are the fastest among transport proteins. They are present in membranes of all organisms from bacteria to higher eukaryotes. Voltage-dependent ion channels (VIC, TC #1.A.1) represent the largest family of channels. Those members thus far functionally characterized are specific for K^+^, Na^+^ or Ca^2+^ ions. There are five *Arabidopsis* VIC members of this family predicted to be chloroplast envelope proteins [29], but none of them have yet been characterized. In the thylakoid membrane, channels from the tandem-pore channel (TPK) subfamily of VIC, from the chloride channel family (CLC, TC #1.A.11), and from the major intrinsic protein family (MIP, TC 1.A.8) have been identified (Fig. 2).

#### The thylakoid K^+^ channel

Using electrophysiology and photosynthetic electron transport measurements, previous reports found evidence for the activity of K^+^ channels in the thylakoid membrane of spinach [39–41]. It has been proposed that K^+^ efflux from the thylakoid lumen is required to balance the light-induced H^+^ pumping across the membrane, thus optimizing photosynthetic activity [42, 43]. In addition, Mg^2+^ and Cl^−^ ions have been suggested to act as counter exchangers for the H^+^ pump [44, 45]. Work describing the preliminary identification of a spinach thylakoid K^+^ channel of 33 kDa was based on western blots with antibodies specific to the pore region in K^+^ channels, anti-KPORE [40]. Electrophysiological studies indicated that it was not voltage gated, but might be ligand-gated, for example, by ATP-dependent phosphorylation. Notably, tetraethylammonium chloride (a general K^+^ channel blocker) only partially inhibited photosynthetic electron transport, which was explained by the possibility that the activity of other ion channels (e.g., Cl^−^) could also contribute to the total ion flux required for charge balance, leading to electroneutral H^+^ pumping and the maximization of photosynthetic activity.

Zanetti et al. [46] identified a putative K^+^ channel gene in the *Synechocystis* genome and named it *SynK*. The amino acid sequence of the corresponding protein contains the selectivity filter for K^+^ transport (amino acid sequence TMTTVGYGD). In the same report, SynK was found to mediate K^+^ transport when expressed in *Escherichia coli* (*E. coli*) mutant strain LB2003, which lacks endogenous K^+^ channels, and to generate a K^+^ selective current when expressed in Chinese hamster ovary cells. Its activity was found to be sensitive to tetraethylammonium chloride and was blocked by cesium (Cs^+^), another general K^+^ channel inhibitor. The structural determinants for the voltage sensitivity have not yet been determined.

Using immunogold electron microscopy, SynK was found to co-localize with *Synechocystis* plasma membranes and with thylakoids [46]. In the same work, *Arabidopsis* TPK3 was localized to the thylakoid membrane. The amino acid sequence of SynK shares 36 % identity with TPK3 from *Arabidopsis*. However, the western blots with anti-KPORE antibody of thylakoid preparations from *Synechocysti*s and *Arabidopsis* indicated that the crossreacting polypeptide bands were of different sizes from the one reported in spinach, namely of 25 kDa for SynK and of 54 kDa for TPK3. Using GFP-based fluorescence microscopy, four *Arabidopsis* TPKs were localized to the tonoplast (TPK1, TPK2, TPK3 and TPK5), whereas TPK4 was found in the plasma membrane [47]. Nevertheless, TPK1, TPK3 and TPK5 harbor putative chloroplast targeting peptides (source: ARAMEMNON). This information, together with the thylakoid localization of TPK3 using western blotting [46], implies that TPK3 and possibly other TPKs have dual subcellular locations, as is the case for other proteins reported in the literature [48, 49]. Interestingly, based on structural differences in the C-terminal domain of TPKs, Dunkel et al. [50] proposed a distinct post-Golgi sorting of TPK1 from the other tonoplast TPKs. Therefore, the detailed mechanisms underlying sorting of plant ion channels still remain to be clarified.

The activity of TPK3 has not yet been characterized in *Arabidopsis*. When expressed in *E. coli* mutant LB2003, *Arabidopsis* TPK1, TPK2 and TPK5 but not TPK3 formed functional K^+^ channels [128]. Nevertheless, TPK3 may function as a K^+^ selective channel in thylakoids mediating K^+^ export from the lumen to the stroma. This flux of positive charge may balance the H^+^ flow, allowing for dissipation of the membrane potential, but not of the pH component of the transthylakoid H^+^ gradient (ΔμH^+^, Fig. 2). This hypothesis has been recently tested in *Synechocystis* by characterization of a SynK knockout mutant [51]. The mutant displayed reduced growth and enhanced sensitivity to high light. Experiments using uncouplers of the pH component of ΔμH^+^ (nigericin) have indicated that the membrane potential was larger in the mutant cells unable to transport K^+^ through SynK. The physiological role(s) in *Arabidopsis* of TPK3 and of other chloroplast-predicted TPKs remain(s) to be investigated.

#### The thylakoid Cl^−^ channel

Chloride channel activities in the thylakoid membrane have been reported in the higher plant *Peperomia metallica* [52] and in the alga *Nitellopsis obtusa* [53, 54]. Movement of Cl^−^ ions into the spinach thylakoid lumen has been reported in the past [45]. Chloride channels are ubiquitous in eukaryotes and prokaryotes and play role in membrane depolarization, regulation of cell volume, resistance to salinity stress and pathogenic response [55]. The chloride channel (CLC) family comprises seven members in *Arabidopsis*, present in various membrane compartments [56]. Although there is no direct evidence for anion currents in *Arabidopsis* thylakoids, one member (CLCe) was found to be targeted to this chloroplast membrane and to be highly expressed in green tissues [57].

Marmagne et al. [57] proposed that ClCe maintains the H^+^ gradient across the thylakoid membrane. The *clce* mutant displayed a photosynthesis-perturbed phenotype, specifically with alterations to the kinetics for chlorophyll fluorescence induction upon transfer to light of dark-adapted leaves [57]. The proposed explanation for this phenotype was a change in the intra-thylakoid ionic status due to a possible defect in anion transport across thylakoids. However, this possibility remains to be investigated. Based on the increased nitrite content in the cytosol of *clce* mutant plants, Monachelo et al. [58] proposed that CLCe most probably transports NO_2_^−^ to compensate for excess positive charge in the thylakoid lumen. This might suggest a role for ClCe in nitrite translocation from the stroma into thylakoids, taking over from the nitrite transporter of the chloroplast envelope [59]. Thus, the link between CLCe and the previous biochemical Cl^−^ channel activities in thylakoids is still missing. The selectivity motif for Cl^−^ transport has not been found in the CLCe amino acid sequence [56], and its anion transport activity has not been characterized either in planta or heterologously, making its function as an anion channel/transporter uncertain.

#### The thylakoid water channel

In chloroplasts, water must be supplied to the thylakoid lumen to sustain photosynthetic water oxidation and for regulatory volume changes of the lumen in the light [60]. The apparent basal water permeability of the spinach thylakoid membrane was found to be low but sufficient to sustain water oxidation. However, thylakoids may harbor water channels (aquaporins) to increase their water permeability, and, thus, to provide net water flow rates suitable for fast regulatory volume changes of the lumen. The values reported in the literature for the activation energy of transport of water into the lumen were as low as those reported for aquaporin-containing membranes, suggesting the presence of water channels in thylakoids. To date, no chloroplast-specific aquaporins have been identified. Large-scale analysis of the chloroplast proteome of *Arabidopsis* indicated that several plasma membrane intrinsic proteins and tonoplast intrinsic proteins (TIP) from the MIP family may be located in chloroplast membranes, but their presence has been regarded as a possible plasma membrane and vacuolar contamination. Specifically, TIP2;1 has been found in thylakoid preparations by two independent research groups [34, 35], whereas TIP1;1 and TIP1;2 were found in envelope preparations [34]. Notably, it has been shown using a heterologous expression system that TIPs mediate not only the transport of water but also of other small solutes such as H_2_O_2_, urea, ammonia and glycerol (reviewed in [61]), implying numerous physiological roles for these proteins in plants. The thylakoid location of TIP2;1 and of other TIPs as well as their potential roles in chloroplasts remain to be demonstrated in dedicated studies. In cyanobacteria, AqpZ is a well-studied water channel, shown to facilitate water transport across the plasma membrane and thus, to play a role in protection against hyperosmotic stress [62]. AqpZ was found using proteomics in *Synechocystis* plasma membranes [37]. However, no aquaporins have yet been identified in cyanobacterial thylakoid membranes.

### Secondary transporters

**Fig. 3:**
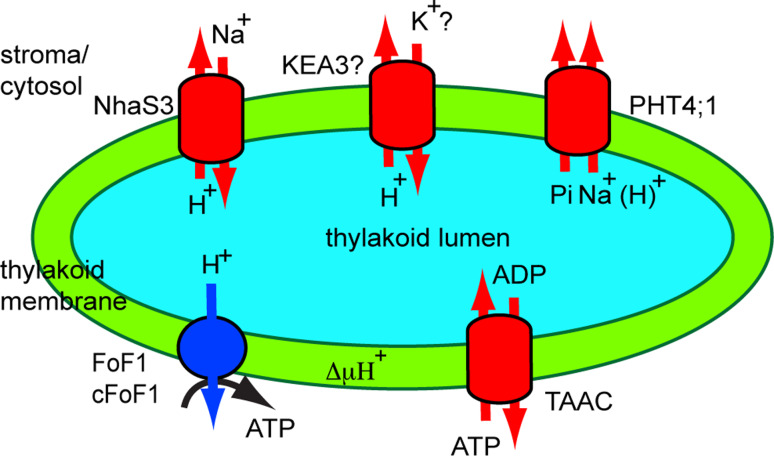
Function of thylakoid secondary transporters. Transporters involved in Na^+^/H^+^ (NhaS3), Pi (PHT4;1) and ATP/ADP (TAAC) exchange are shown in the thylakoid. TAAC supplies stromal ATP in the plant thylakoid lumen for nucleotide-dependent reactions. Pi resulting from these reactions is exported back to the stroma by PHT4;1 whose activity is driven by electrochemical potential generated by exchangers such as plant homologues of NhaS3. A K^+^/H^+^ exchanger KEA (KEA3) may also be located in the thylakoid and uses ΔμH^+^ to import K^+^ ions into the lumen

Secondary or electrochemically-driven transporters (TC #2) work using the concentration gradient of co-transported molecules. The major facilitators (MFS, TC #2.A.1) represent the largest secondary transporter family in all organisms and transport a wide variety of substrates, including carbohydrates, inorganic phosphate (Pi), amino acids and cations. Protons and Na^+^ ions are used as co-transported molecules by 80 % of the MFS members. Transporters belonging to MFS, but also to the mitochondrial carrier family (TC #2.A.29) and to the monovalent cation:proton antiporter families (CPA1, TC #2.A.36 and CPA2, TC #2.A.37) have been identified in the thylakoid membrane (Fig. 3).

#### The thylakoid Pi transporter

Pi transporters are essential for chloroplasts since they control the level of Pi in the stroma and the homeostasis required to initiate the CO_2_ fixation cycle [63]. The most recently characterized Pi transporters are known as PHT4s (formerly annotated as anion transporters, ANTRs [64]), and belong to the anion:cation symporters (TC #2.A.1.4) within MFS. The PHT4 family shares significant sequence similarity with the solute carrier 17 family (SLC17) of mammalian anion transporters (TC # 2.A.1.14.13) [64]. *Arabidopsis* has six PHT4s, which, when expressed in *Saccharomyces cerevisiae* (*S. cerevisiae*), were shown to specifically transport Pi in an H^+^-dependent manner [65]. The rates of Pi transport in yeast were considerably inhibited by the addition of protonophore carbonylcyanide m-chlorophenylhydrazone (CCCP), suggesting that transport may be dependent on ∆μH^+^, although secondary effects, such as changes in cytosolic pH, may also have affected the transport activity. The specificity for Pi of PHT4;1 transport was demonstrated independently by another laboratory [66]. However, its transport activity was found to be dependent on an electrochemical Na^+^ gradient when assessed across the *E. coli* membrane. It was therefore proposed to be electrogenic. In the same report, the most efficient competitors in bacterial cells transformed with recombinant PHT4;1 were nonlabeled Pi and its analogue, methylphosphonate. The fact that glutamate competed by 56 % with the Pi uptake in bacterial cells indicated that PHT4;1 could bind, but not necessarily transport this organic anion. Pre-incubation with the Na^+^ ionophore monensin reduced P_i_ accumulation into transformed cells. Site-directed mutagenesis of PHT4;1 and functional characterization in *E. coli* has revealed important charged residues for the binding and translocation of Pi and Na^+^ dependency, which are fully conserved in all PHT4 members and partially conserved in SLC17 members [67].

PHT4;1 was initially localized to the chloroplast using GFP fluorescence microscopy [64]. Later on, it was more precisely localized to the thylakoid membrane using peptide-specific antibodies [66]. A proteomics report indeed found the protein in chloroplast preparations, but it was assigned to the envelope membrane since it could not be detected in thylakoid preparations [34]. In support of a thylakoid location, the *PHT4;1* gene was found to be mainly expressed in photosynthetic tissues and displaying a circadian rhythm-regulated expression pattern (with a peak during the light) [68].

The transport activity of PHT4;1 in terms of the direction of transport, and whether it is driven by either H^+^ or Na^+^ gradients across thylakoids, has not yet been investigated in *Arabidopsis*. Another PHT4 member localized to root plastids, PHT4;2, has been characterized as a Na^+^-dependent transporter [69]. In plants, PHT4;1 has been proposed to export Pi generated during nucleotide-dependent reactions in the thylakoid lumen to the stroma [70]. Initial analyses of its physiological role indicated that a *pht4;1* knockout mutant displayed reduced growth, lower photosynthetic electron transport rates and faster induction of photoprotective mechanisms during high light stress [71]. The effects were not dramatic, suggesting an indirect influence on photosynthesis, possibly via electrogenic transport, as indicated by its analysis in *E. coli*. The possibility of faster acidification of the thylakoid lumen in the mutant as compared to wild type plants remains to be investigated. Interestingly, an *Arabidopsis* mutant lacking PHT4;1 has been studied in a screen for suppressors of an accelerated cell death mutant [72]. The *pht4;1* mutant was found to be susceptible to infection with virulent *Pseudomonas syringae*, but treatment with a salicylic acid agonist induced resistance in this mutant, indicating that PHT4;1 acts upstream of the salicylic acid pathway. It was, therefore, proposed that PHT4;1 is involved in regulating innate immunity in *Arabidopsis*, but the relevance of its Pi transport activity remained unclear in this context. Defense signaling networks are complex and may involve crosstalk to many other signaling pathways, such as the one in the chloroplast thylakoid membrane, possibly related to nucleotide metabolism in the thylakoid lumen.

#### The thylakoid ATP/ADP carrier

The mitochondrial carriers are proteins only found in eukaryotic cells. The first members of this family were recognized in the mitochondrial inner membrane. The most studied members are the mitochondrial ADP/ATP carriers (AAC, TC #2.A.29.1.1). There are at least five putative mitochondrial AACs in *Arabidopsis*, but only some of them have been characterized [73–75]. In addition to the mitochondrion, AAC members have been also found in peroxisomes, hydrogenosomes, amyloplasts and chloroplasts [73, 76].

Using western blotting and activity inhibition with an antibody against a bovine mitochondrial AAC, the activity of an AAC was reported in the spinach thylakoid membrane [77]. Notably, the molecular weight in SDS-PAGE of this protein was much higher than that of a typical AAC (36.5 versus 28 kDa). BlastP searches using the bovine AAC sequence in the *Arabidopsis* database revealed two chloroplast candidates for proteins responsible for this transthylakoid activity, encoded by the *At3g51870* and *At5g1500* genes and having a theoretical mass of 38.5 kDa for the processed forms. Based on findings of its initial localization and functional characterization in *E. coli,* the latter one was annotated as the thylakoid ATP/ADP carrier (TAAC) [78].

*Arabidopsis* TAAC shares about 30 % identity with classic AACs, which is concentrated in the six putative transmembrane helices and to a lower degree in the connecting loops [78]. However, the amino acid sequence of the processed form is 80 residues longer than that of a typical AAC, explaining the difference in the reported size in SDS-gels [77]. The extra 80 residues are distributed as 50 in the N-terminus and 30 in the C-terminus, regions containing many charged residues and a five-glycine repeat, which could play a role in the regulation of TAAC activity.

*Arabidopsis* TAAC was characterized in *E. coli* as an ATP importer in exchange for ADP [78, 79]. This function was validated in the same report by transport assays using *Arabidopsis* thylakoids from wild type plants and a *taac* mutant. TAAC transport activity shows similarities but also differences from the activity of mitochondrial AACs. For example, the addition of protonophore carbonylcyanide-4-trifluoromethoxyphenylhydrazone (CCCP) in transport assays performed in *E. coli* increased the apparent *K*_m_ of TAAC for ATP but not for ADP, whereas the corresponding *K*_m_ values were only slightly lowered by CCCP in the case of mitochondrial AACs expressed in *E. coli*. Also, the export of ADP by TAAC was found inhibited by CCCP at variance with the same activity performed by the mitochondrial AACs [73]. The mechanistic reasons for these discrepancies with respect to CCCP are not known.

TAAC was localized using GFP fluorescence microscopy to the chloroplast and most recently also to root plastids [78, 79]. Within the chloroplast, TAAC was found to have a dual location, namely in the thylakoid membrane and in the envelope inner membrane, based on immune gold labeling, western blotting with peptide-specific antibodies, and GFP fluorescence microscopy [78, 79]. Subfractionation experiments indicated the stroma-exposed lamellae as the precise location of TAAC within the thylakoids [78]. A previous proteomics report confirmed the chloroplast location of TAAC [35]. Nevertheless, it was classified exclusively as an envelope protein by another report [34]. One plausible explanation for detection of only low levels of TAAC in the analyzed thylakoid preparation may be a considerable loss of the stroma-exposed lamellae, where TAAC is located within thylakoids. The integrity and purity of thylakoid membranes in these preparations remains to be assessed by western blotting for markers of the stroma thylakoid regions. Three different methods support a dual location of TAAC, and, thus, provide strong arguments against the restricted envelope proposed by proteomics studies. The protein encoded by the *At3g51870* gene was only detected in envelope preparations by both proteomics and western blotting with peptide-specific antibodies ([34] and Spetea, unpublished observations).

Supporting a dual location, TAAC was found expressed in both photosynthetic (containing thylakoids) and non-photosynthetic (thylakoid-free) organs. Moreover, it was found highly expressed in etiolated and young seedlings, young organs, mature plants and growing tissues [78, 79]. This expression pattern could indicate a role in biogenesis and developmental processes requiring adenine nucleotides. Moreover, TAAC expression is strongly upregulated in leaves undergoing senescence or exposed to wounding, light stress, oxidative stress, salt stress and desiccation [78], pointing to additional physiological roles of TAAC.

Via adenine nucleotide exchange in the thylakoid membrane, TAAC was proposed to supply ATP for nucleotide-dependent reactions in the thylakoid lumen [77]. A recent model for its function during PSII repair cycle under high light stress has been presented [70]. Briefly, ATP translocated by TAAC into the lumen is either used as such in phosphorylation reactions or is converted to GTP by the lumenal nucleoside diphosphate kinase, and GTP is then bound to the PsbO lumenal extrinsic subunit of the PSII dimeric complex [77, 80]. The GTPase activity of this protein regulates the monomerization and partial disassembly of PSII, pre-requisite steps for the proteolytic degradation of the reaction center D1 protein [81]. This model has been validated by the inability of the *taac* mutant to degrade the D1 protein, making it highly sensitive to high light stress [82]. The identification of a lumenal phosphatase and of phosphorylated proteins known to be involved in PSII repair point to a role for the ATP supplied by TAAC into the lumen in the assembly steps of the cycle. The kinase responsible for phosphorylation of lumenal proteins remains to be identified. In addition to its role in PSII repair, TAAC (like PHT4;1) appears to influence thermal photoprotection during high light stress [82]. This effect could be explained by the fact that its thylakoid transport activity may be electrogenic and driven by ∆μH^+^ across the thylakoid membrane, as was found in *E. coli* [78].

Recently, Palmieri et al. [83] predicted novel substrates for transport by TAAC, namely Coenzyme A and phosphoadenosine phosphate (PAP). Moreover, TAAC is co-expressed with genes related to metabolism of sulfated secondary metabolites (glucosinolates). Gigolashvili et al. [79] revisited the transport activity of TAAC and found that recombinant TAAC produced in either *E. coli* or *S. cerevisiae* cells and reconstituted in proteoliposomes could function as an exchanger for 3′-phosphoadenosine 5′-phosphosulfate (PAPS) and either PAP or ATP [79]. ADP was a much less preferred substrate, and AMP was not transported at all [79]. The leaves of *taac* mutants displayed reduced levels of glucosinolates and also of sulfated peptides as compared to the wild type. Therefore, a role for TAAC in sulfur metabolism was proposed. Interestingly, PAP produced inside the chloroplast has been reported to be a component of a signaling pathway from the chloroplast to the nucleus (retrograde signaling) that could alter nuclear gene expression for chloroplast biogenesis, homeostasis, or abiotic stress response [84]. Since PAP was found to be a substrate for TAAC, the envelope-located protein has been proposed to mediate the flux of metabolic retrograde signals [85]. Thus, based on the dual location and types of substrates used for transport, the role of TAAC in *Arabidopsis* appears to be more complex than originally proposed.

#### The thylakoid Na^+^/H^+^ antiporter

Na^+^/H^+^ antiporters influence H^+^ or Na^+^ ion motive force across the membrane in response to environmental changes. Such a protein, named NhaS3, has been localized in *Synechocystis* to the thylakoid membrane [86]. Among the five NhaS antiporters in *Synechocystis,* NhaS3 is thought to be essential for cell viability, since a mutant affected in this gene could not be obtained [87]. NhaS3 displayed expression levels higher than, and could functionally replace the other four antiporters, emphasizing its great importance for *Synechocystis*.

When expressed in *E. coli*, NhaS3 was found to import Na^+^ in exchange for H^+^ [88], results confirmed later by Tsunekawa et al. [86], who also showed that this activity was independent of pH in the range 6.5-9.0. Moreover, it was found that NhaS3 elevated K^+^ uptake into *E. coli*. Since NhaS3 did not display K^+^/H^+^ exchange activity, it was proposed to contribute to maintaining a balance in the Na^+^/K^+^ ratio. Tsunekawa et al. [86] also proposed that NhaS3 may transport Na^+^ into the lumen in exchange for H^+^, and, thus, may fulfill two functions, namely to reduce the toxic effects of Na^+^ in the cytosol during salt stress, and of the acidic pH in the lumen. However, the authors of that report did not test the toxic effects of Na^+^ on the activity of PSI and PSII in terms of O_2_ evolution and electron transport. Since Allakhverdiev et al. [89, 90] reported inactivation of these complexes by salt stress, the conclusions of [86] must be revisited.

Na^+^ transported into the lumen may be used to drive Pi export by PHT4;1 in *Arabidopsis,* as proposed by Pavon et al. [66]. However, an *Arabidopsis* thylakoid homologue to NhaS3 has not been identified thus far. Instead, an envelope homologue CXH23 was reported in *Arabidopsis* [91]. Plastids from *chx23* mutants had straight thylakoids but lacked grana structures. Furthermore, *chx23* mutants displayed a high sensitivity to NaCl. CHX23 has been recently proposed to work as a chloroplast Na^+^(K^+^)/H^+^ exchanger important for potassium and pH homeostasis and chloroplast development and function [92].

#### The thylakoid K^+/^H^+^ antiporter

As transport systems for K^+^ across the *Arabidopsis* thylakoid membrane, only the K^+^ channel TPK3 has been thus far identified [46]. It is thought to mediate K^+^ export from the thylakoid lumen. This implies that a transport system for the import of K^+^ into the lumen must exist. Interestingly, this channel has not yet been identified by proteomic analyses of *Arabidopsis* chloroplast membranes, which instead indicated three members of the K^+^ exchange antiporter subfamily (KEA), belonging to CPA2, namely KEA1, KEA2 and KEA3 [34]. These are the chloroplast-predicted proteins of the six-member KEA family in *Arabidopsis* (source: ARAMEMNON). KEA1 and KEA2 were clearly identified as envelope proteins in proteomic studies [34, 93], whereas KEA3 was found in the thylakoid membrane [35]. KEAs have been proposed to transport K^+^ into acidic compartments against an electrical gradient and to be driven by the H^+^ pumps of these compartments [94]. Indeed, when expressed in yeast and reconstituted in proteoliposomes, KEA2 mediated K^+^/H^+^ exchange in a manner dependent on an imposed pH gradient (acidic inside) [95]. Dedicated studies are required for the localization of chloroplast-predicted KEAs and for their involvement in chloroplast function.

### Primary transporters

**Fig. 4:**
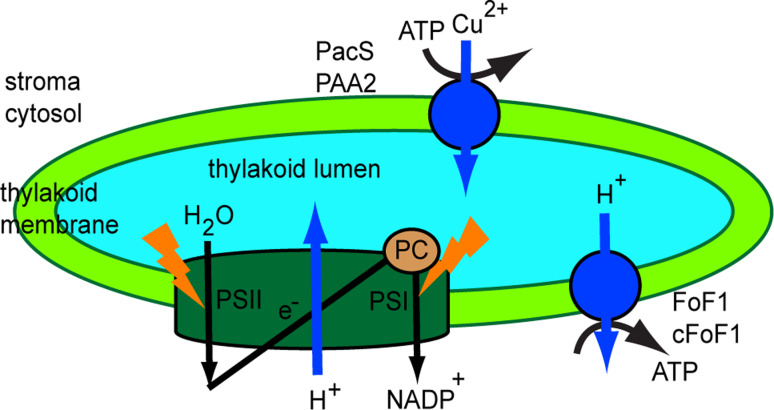
Function of the thylakoid Cu^2+^-transporting ATPase. *Synechocystis* PacS and *Arabidopsis* PAA2 are located in the thylakoid and use energy from ATP hydrolysis to supply Cu^2+^ to plastocyanin (PC), an essential cuproenzyme of the photosynthetic machinery

Primary transporters directly use energy to transport molecules across membranes. Most of the enzymes that perform this type of transport are transmembrane ATPases, which are of the ABC-, F-, V-, and P-type. In the thylakoid membrane, one F-ATPase (TC #3.A.2) and one P-ATPase (TC #3.A.3) have been identified thus far (Fig. 4). The knowledge about the H^+^-translocating ATP synthase (F_0_F_1_), which is an F-ATPase, has been reviewed by Junge et al. [96] and by Seelert and Dencher [97].

#### The thylakoid Cu^2+^-transporting ATPase

Photosynthetic machinery is abundant in metal-containing proteins, such as plastocyanin located in the thylakoid lumen. The transport of Cu^2+^ ions across pea thylakoid membranes was previously reported [98]. As indicated by experiments employing various types of inhibitors, neither uniporters nor antiporters mediated this activity, and a transthylakoid H^+^ gradient was not required. The same report proposed that there must be more than one transporter for copper, since Zn^2+^ inhibited Cu^2+^ transport to a maximum of 60 % [99].

The P-ATPase family includes the heavy metal P1B-ATPase (HMA) family, which translocates cations across biological membranes using energy from ATP hydrolysis. Among the 46 P-ATPases predicted in *Arabidopsis* [100], three have been identified in the chloroplast inner envelope membrane, namely the Cu^2+^-ATPase PAA1 (HMA6) [101], the Cu^2+^, Ca^2+^ and Zn^2+^-ATPase HMA1 [102] and the autoinhibiting Ca^2+^-ATPase ACA1 [103]. One has been found in the thylakoid membrane, namely the Cu^2+^-ATPase PAA2 (HMA8) [104]. A direct link between the previously described Cu^2+^ transport activity and PAA2 is still missing since its biochemical activity has not yet been characterized.

An analysis of *paa1* knockout mutants revealed a deficiency in photosynthetic electron transport, likely due to impaired import of Cu^2+^ ions into the chloroplast [101]. The *paa2* mutant had a similar, though not as severe, phenotype, but the double *paa1*-*paa2* mutant was seedling lethal [104]. Based on these findings, PAA1 and PAA2 transport copper into the chloroplast and across the thylakoid membrane, supplying the lumen with this cofactor for plastocyanin, a copper-binding protein involved in electron transport between cytochrome cytb_6_f and PSI.

In cyanobacteria two Cu^2+^-transporting P-type ATPases have been identified, namely PacS located in the thylakoid membrane [105] and CtaA located in the cytoplasmic membrane [106]. These proteins have been shown to supply copper to plastocyanin for photosynthesis in the same way as PAA1 and PAA2. Based on yeast two hybrid interactions and NMR, a Cu chaperone antioxidant protein (Atx1) has been proposed to take Cu^2+^ ions from CtaA or other sources and deliver it to PacS [107, 108]. The importance of Atx1 for Cu^2+^ homeostasis and conferring tolerance to both excess Cu^2+^ and Cu^2+^ deficiency was recently demonstrated [109]. Tapken et al. [110] reported that the stability of the PAA2 protein is affected by Cu availability, more specifically it decreases when plants are grown with elevated Cu^2+^ and increases in *paa1* mutants. It was therefore proposed that plastocyanin, which is the target of PAA2, is involved in this regulatory mechanism.

## Evolutionary origin of thylakoid channels and transporters

Much is known about channels and transporters located in the chloroplast outer and inner envelope membranes [63, 111, 112]. Comprehensive analysis of the *Arabidopsis* chloroplast envelope transportome has indicated that the majority of these proteins are of host origin, driven by the requirement to establish communication via the exchange of metabolites between the host cytosol and the cyanobiont [113]. Thylakoid channels and transporters, on the other hand, may have either a host or a cyanobiont origin. We have examined the phylogenetic evidence for the origin of each transport protein family reviewed above, focusing on the thylakoid-located members.

**Fig. 5:**
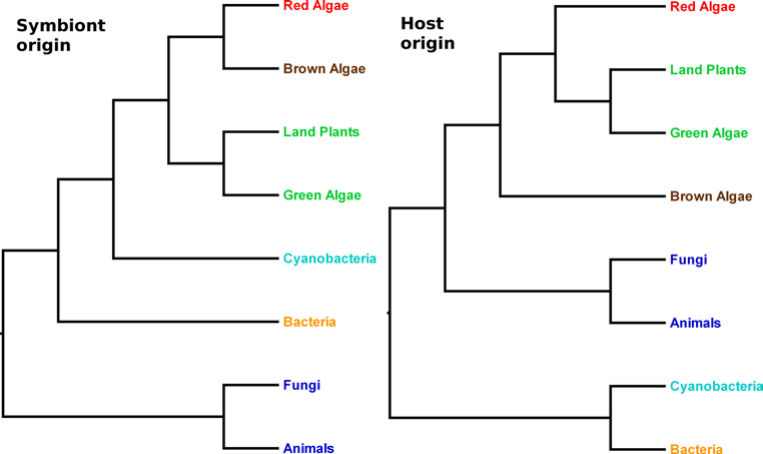
Hypothesis for the evolutionary pathways of thylakoid transporters. (*Left panel*) Symbiont origin proteins in Archaeplastida (green plants, red algae and lineages that have derived their chloroplasts from red algae) are expected to be more closely related to cyanobacterial lineages than to non-Archaeplastida, such as animals and fungi. (*Right panel*) If the proteins have a host origin, then a closer relationship between Archaeplastida lineages and other eukaryotes, such as animals and fungi, is expected. In a symbiont origin phylogeny, brown algae derive these proteins via red algae, and are expected to be more closely related to that lineage

No homologues of the thylakoid transporters thus far described in *Arabidopsis* and *Synechosystis* have been described in algae, although similar sequences can be recognized in many of the sequenced genomes. Phylogenetic analyses show that the *Arabidopsis* channels and transporters can be divided into two groups. In the first group are those proteins whose origins appear to be derived from cyanobacterial lineages (Fig. 5: *left panel*), and would, therefore, have been present in the common ancestor of Archaeplastida (which includes glaucophytes, red algae and green plants) at the time of the cyanobiont acquisition. These proteins include two members of the HMA family (PAA1 and PAA2; Supplemental Fig. S1), and three members of the KEA family (KEA1-3; Supplemental Fig. S2). In the second group are those transport proteins whose closest relatives are instead found among eukaryotes other than Archaeplastida. They would, therefore, appear to have been co-opted from existing gene families present in the common ancestor of most or all eukaryotes (Fig. 5: *right panel*). In *Arabidopsis*, this group includes five members of the PHT4 gene family (PHT4;1 to PHT4;5: Supplemental Fig. S3), both members of the TAAC family (TAAC and the protein encoded by the *At3g51870* gene [3]), the TPK family (TPK1 to TPK5; Supplemental Fig. S4) and the TIP family [114]. For the remaining family of thylakoid proteins reviewed here (CLC), the phylogenetic results do not decisively show their evolutionary origins (Supplemental Fig. S5). The 28-member CHX family in *Arabidopsis* does not include any known thylakoid-located members (only a chloroplast envelope one [91]), but is also mentioned below with respect to its relationship to the KEAs.

### Symbiont origin

Of those transport proteins with an origin among cyanobacterial lineages, some are found in many groups of plastid-bearing organisms and may be of critical importance to photosynthetic functions, whereas others appear to have been lost in some descendent lineages and, thus, may not be as important.

#### The thylakoid Cu^2+^-transporting ATPase

PAA1 and PAA2, located in the chloroplast inner envelope [96] and thylakoid [99], respectively, share a common ancestor prior to the divergence of the green alga *Chlamydomonas* and land plants, and are derived from the same origin (Supplemental Fig. S1). A host origin is rejected, because the phylogenetic analysis excludes the closest BlastP matches of non-Archaeplastida eukaryote lineages from the clade that groups only cyanobacteria, bacteria and green plants. Although this tree places a bacterial lineage closer to the cyanobacteria (instead of green plants and other Archaeplastida occupying this position, as shown in Fig. 5 *left panel*), this could have been caused by horizontal gene transfer, which has been previously observed between cyanobacteria and other bacteria [115]. It is clear from the phylogeny (Supplemental Fig. S1) that the thylakoid PacS protein from *Synechocystis* is not orthologous to the thylakoid PAA2 protein from *Arabidopsis*, since the gene lineages that produced these proteins had already diverged in the common ancestor of these two taxa. What is not certain is whether the original location of the protein that gave rise to PAA1 and PAA2 was in the envelope or in the thylakoid. A shift from one location to the other, presumably made possible by the ancient gene duplication, would be supported if the protein orthologues found in *Oryza,* *Physcomitrella* and *Chlamydomonas* (marked with red branches in Supplemental Fig. S1) and other green plants not sampled here, were localized to the same place as each PAA copy to which they are most closely related. No orthologues of PAA1 or PAA2 were found by BlastP searches in either red algae or glaucophytes, suggesting that these lineages may have lost the genes that encode these proteins.

#### The thylakoid K^+/^H^+^ and Na^+/^H^+^ antiporters

KEAs resemble the bacterial K^+^/H^+^ antiporters KefB and KefC [116]. Interestingly, detailed phylogenetic analyses of KEAs indicated that the chloroplast-predicted KEAs (KEA1, KEA2 and KEA3) share a most recent common ancestor with the CHX subfamily of CPA2, including CHX23 [94]. Our phylogenetic analysis does not include the CHX subfamily, but a broader analysis (not shown) adds all CHX proteins as an additional clade sister to the rest of those proteins shown in Supplemental Fig. S2. Although at first glance this would appear to be contradicting the earlier result [94], the mid-point rooting used in our trees should not be taken for granted. The root could also lie along the stem lineage of the KEA1 to KEA3 clade (that includes *Gloeobacter* glr1343 KefC), the KEA4 to KEA6 clade (that includes *Ectocarpus* CBJ26612), the CHX clade (not shown), or the clade of four cyanobacterial proteins (that includes *Synechocystis* NhaS3). A rooting along the stem KEA4 to KEA6 lineage would, for example, imply a closer relationship between KEA1 to KEA3 and the CHX subfamily, as found previously [94].

Irrespective of the four most reasonable root locations described above, we found that the NhaS3 protein from *Synechocystis* is not orthologous to any of the six *Arabidopsis* KEAs. A better candidate among cyanobacteria for the orthologue to KEA1 to KEA3 would be the *Gloeobacter* KefC protein, although the phylogeny doesn’t unambiguously support this either, because of the closer relationship of non-cyanobacterial bacterial lineages to these KEAs. There may have been at least five distinct gene lineages producing K^+^ and Na^+^ antiporters present in the common ancestor of bacteria and eukaryotes (a single gene lineage represented by the clades including *Synechocystis* ZP21042641, *Synechocystis* NhaS3, *Gloeobacter* gll0383, and two or more gene lineages for the clade including *Gloeobacter* KefC and *Arabidopsis* KEA1 to KEA3). Not all of these gene lineages appear to have remained in all descendents. It could be that partitioning and/or overlap of functions among the proteins derived from non-orthologous lineages has occurred. If this was the case, then we would expect to find cases of convergent evolution among non-orthologous lineages as well as the loss of redundant gene lineages in some cases.

The six KEA proteins found in *Arabidopsis* are distributed across three lineages (Supplemental Fig. S2; [117]). The envelope-located KEA1 and KEA2 are very closely related to each other, and are most closely related to a bacterial protein (of non-Archaeplastida proteins), and, therefore, both would appear to have a symbiont origin, although a cyanobacterial representative is lacking in this clade. The thylakoid-located KEA3, on the other hand, appears to have been acquired separately. The origin of KEA3 is less certain, because neither cyanobacterial, nor bacterial, nor non-Archaeplastida sequences among the best BlastP matches in various searches were found to be members of the clade that includes KEA3. However, the very weak support for the branch grouping KEA1+ KEA2 and KEA3 means that a placement of the KEA3 clade among other bacterial lineages in the KEA tree is also possible. Furthermore, the clade containing KEA1 to KEA3, cyanobacterial and several bacterial lineages is strongly supported and excludes all non-Archaeplastida best BlastP matches to these KEAs (the cryptomonad and heterokont proteins included here notwithstanding—all of which have gained chloroplasts via secondary endosymbiosis of Archaeplastida ancestors [118]. Thus, a symbiont origin is also most likely for KEA3. Slightly complicating this finding is the stronger support for KEA1+ KEA2 and KEA3 found recently [117], which raises the possibility that these proteins share their origin. But in any case, the strongly supported clade with all three of these proteins is consistent with our results above. Thus, both phylogenies of the KEAs find a cyanobacterial origin as the most plausible. No orthologues of KEA1 to KEA3 were found in glaucophytes in BlastP searches, but a probable orthologue to KEA3 was found in the brown alga *Ectocarpus* (CBN80227), along with a closely related but probable paralogue to KEA3 in the red alga *Galdieria* (Supplemental Fig. S2).

KEA4, KEA5 and KEA6 are also very closely related to one another (Supplemental Fig. S2), but it is unclear whether they have a symbiont origin along with the aforementioned KEAs. Although cyanobacteria are found sister to the green plant clade containing these KEAs, this relationship is not robustly supported (posterior probability <0.95). These proteins are all strongly predicted to the secretory pathway in *Arabidopsis* (source: ARAMEMNON), which is more consistent with a host origin of this protein lineage.

### Host origin

Among those proteins with a host origin, only TAAC and PHT4;1 have homologues in green plants (green algae + land plants). This could be explained by the similarity of their pigment-protein composition and thylakoid organization compared to cyanobacteria, red and brown algae (Fig. 1). These two transporters (TAAC and PHT4;1) may have evolved in response to the demands of new activities found in green plants involving nucleotide-dependent reactions in the thylakoid lumen [70]. Two other protein families (TIP and TPK) may have host origins, although the evidence is weaker in these cases.

#### The thylakoid ATP/ADP carrier

The phylogeny of TAAC has been previously presented [3] and the results are briefly summarized here for completeness. TAAC has orthologous sequences in green algae as well as in many sequenced land plants. It appears to have evolved via gene duplication from a pre-existing gene family prior to the diversification of these taxa, but after they separated from other eukaryotes. A second TAAC-like protein found in *Arabidopsis*, whose location is most likely in the envelope [34], was derived from gene duplication after the eudicots diverged, but earlier than the origin of the family Brassicaceae. The relatively recent origin of TAAC (in a green plant ancestor) suggests that it has a specialized function, perhaps associated with the unique thylakoid organization in this lineage [3]. This function could be related to the need for highly controlled regulation of PSII repair in a highly stacked thylakoid membrane system (Fig. 1) that requires migration of complexes from appressed to non-appressed regions. Additional specialized functions for TAAC could be those reported in the envelope, namely of a PAPS transporter with role in sulfur metabolism [79] and of a PAP transporter with postulated role in retrograde signaling during chloroplast biogenesis and abiotic stress response [85].

#### The thylakoid Pi transporter

BlastP searches using any of the *Arabidopsis* PHT4 sequences at NCBI have failed to find homologues in cyanobacteria, brown algae, diatoms or glaucophytes. The lack of alignable cyanobacterial sequences and the presence of alignable non-Archaeplastida eukaryotes in the phylogeny (Supplemental Fig. S3) clearly exclude a symbiont origin for the gene family that encodes these proteins. Nevertheless, 35–70 % similar sequences could be found in red algae, green algae and land plants, indicating an early origin within the Archaeplastida, which is confirmed by the deep position of the red alga sequence in the phylogeny, although perhaps after the glaucophytes diverged from the remaining Archaeplastida lineages.

PHT4;1, however, diverged from its closest paralogue within a land plant lineage (and, hence, more recently than TAAC) as the product of a gene duplication in the common ancestor of flowering plants, sometime after the divergence of both the spike mosses and mosses from this lineage. However, this final divergence between a clade containing the thylakoid-located PHT4;1 and a clade containing the chloroplast envelope-located PHT4;4 [64] is just one of several gene duplications that have added to the diversity among PHT4 gene family members in *Arabidopsis*. The earliest division separated a clade containing the Golgi-located PHT4;6 [65] and the remainder. The next division produced a lineage including PHT4;5 found in chloroplasts [65] from the remaining members. The following division produced two lineages, one containing PHT4;2 and PHT4;3, found in the root plastid envelope [69] and probably chloroplasts (source: ARAMEMNON), respectively, and the other containing the PHT4;1 and PHT4;4 copies.

One PHT4-related protein has been found in the red alga *Cyanidioschyzon*, but it is unclear from the phylogeny whether this copy belongs to the PHT4;2 + PHT4;3 or the PHT4;1 + PHT4;4 lineage. In any case, the PHT4;5, PHT4;6, PHT4;2 + PHT4;3 and PHT4;1 + PHT4;4 lineages include representatives from the green alga *Chlamydomonas* and together with the presence of the red algal representative, suggest that the earliest two or three divisions of this protein family date back to prior to the division of red algae from green plants. If the Golgi location of PHT4;6 represents the original location (which is also consistent with a host origin), then the specialization of plastid-located members places a lower (younger) boundary on the age of this split, namely the origin of plastids. PHT4 proteins in general, therefore, may not necessarily have specialized chloroplast functions in Archaeplastida, unlike the case of TAAC [3]. However, the thylakoid-located PHT4;1 member, whose location we hypothesize was shifted to the thylakoid membrane comparatively recently, could well have a highly specialized and evolutionarily novel function in this location.

#### The thylakoid water channel

Phylogenetic analyses indicated that, compared to the large and ancient family of MIPs, that has members in all living organisms, TIPs evolved in the lineage leading to higher plants [119]. A previous phylogenetic study [114] suggested that these proteins have a host origin, being grouped with many non-Archaeplastida eukaryote aquaporins, although the critical branches did not receive strong support. However, the most closely related cyanobacterial lineages to the TIPs are strongly grouped with other bacterial lineages (and exclude the TIPs), which is consistent with a host origin (as in Fig. 5: *right panel*). Although all TIPs are found in the tonoplast, presumably indicating their original location, three members could also be found in chloroplast membranes (TIP1;1 and TIP1;2 in the envelope, and TIP2;1 in the thylakoid) [34, 35]. These proteins are found in two separate branches in the phylogeny, indicating independent transfers to their new chloroplast locations, perhaps as recently as in the stem of the flowering plant lineage [119].

#### The thylakoid K^+^ channel

The phylogeny (Supplemental Fig. S4) indicates that TPK proteins appear to have a host origin, but the support for the grouping of plant and other eukaryote taxa in the phylogeny is weak. However, the most closely related cyanobacterial lineages to TPK3, including SynK, are strongly grouped with other bacteria, which potentially rules out a closer relationship between them and green plants (although horizontal transfer cannot be excluded). On balance, we tentatively suggest that TPK proteins may have a host origin.

Six related TPKs are present in *Arabidopsis*. These proteins diversified into two lineages prior to the split between mosses and flowering plants, but after the brown algae—green plant divergence. If a host origin is correct, this would suggest that brown algae have derived their TPK orthologues via the host genome, and that the other Archaeplastida lineages have lost TPK orthologues. The possible dual location of some of these proteins mentioned previously makes any inference of the original location of this protein lineage difficult, although a recent shift (after the monocot–eudicot divergence) to the thylakoid of TPK3 is suggested.

### Unknown origin

#### The thylakoid Cl^−^ channel

Phylogenetic analysis of the CLC family indicated that the CLCe sequence is highly similar to bacterial CLCs, shown to function as H^+^/Cl^−^ antiporters [56, 57]. For CLCe, some cyanobacterial homologues have been identified using bioinformatics [57], but their function remains to be investigated. BlastP at NCBI indicated the presence of homologues in green algae, brown algae, diatoms and cyanobacteria, sharing 25-35 % identity with CLCe, but thus far none have been characterized. As mentioned above, the phylogenetic results (Supplemental Fig. S5) cannot distinguish between a host or symbiont origin for ClCe, although a split between the ClCe and ClCf copies in *Arabidopsis* appears to be younger than the land plant—green algae divergence, but still very early in land plant evolution.

To summarize, thylakoid transporters can be classified into those of symbiont origin and those of host origin (Fig. 5). However, within these categories some differences arise. Among proteins of symbiont origin, it is unclear whether the thylakoid location of PAA2 is ancient or relatively recent, and, thus, whether this protein lineage has become recently specialized for thylakoid function, or not. However, the lack of orthologous proteins in red algae and glaucophytes suggests either a non-essential function of PAA2 (and probably an ancient presence in the thylakoid, with losses in those lineages) or a specialized function specific to green plants (and thus a more recent shift to the thylakoid, without losses in the other lineages of thylakoid-located members). Testing whether the function of PAA2 is somehow predicated upon the green plant thylakoid structure may go some way to distinguishing among these alternatives.

KEA3, although also ultimately of symbiont origin, has been derived very early, as a putative orthologue in the brown alga *Ectocarpus* has been identified in the phylogeny. This suggests that this protein lineage may have been present in thylakoids a long time, especially if the location of the *Ectocarpus* protein was also in thylakoids. If so, this would suggest that KEA function in thylakoids is an old one, but perhaps not critical, as we could not find additional orthologous protein sequences among red algae or glaucophytes, for example, suggesting several losses of this protein lineage. Characterization of the *Ectocarpus* protein is thus an important research question, as is discovering whether and how red algae and cryptophytes transport K^+^ and Na^+^ into their thylakoids.

Among host origin proteins, recent or ancient origins of thylakoid location are also possible. Both PHT4;1 and TIP2;1 appear to have relatively recent origins in thylakoids. In PHT4;1, this protein arose via gene duplication from an existing plastid-located lineage, which itself was derived from a host (and therefore non-plastid located) protein family. The duplication that gave rise to PHT4;1 probably occurred somewhere on the stem of flowering or seed plants and is a quite recent shift to the thylakoid. TIP2;1 may have arisen via gene duplication at a similar time, although the sampling and the resolution in the PHT4 and TIP phylogenies, respectively, are insufficient to place these events definitively.

TAAC also appears to have a relatively recent origin of thylakoid location, because the clade of TAAC-like proteins include only green plant members, and diverged from another clade containing only green plant members via gene duplication [3]. Thus, TAAC probably obtained its thylakoid location in the stem green plant lineage, clearly earlier than PHT4;1 and TIP2;1, but again, comparatively recently in relation to the origin of plastids. The most recent shift to the thylakoid among proteins reviewed here, however, seems to have occurred in TPK3 after the monocot—eudicot divergence, although this conclusion could be challenged if additional locations of other TPKs are subsequently demonstrated.

## Methods

### Sequence selection

We began with sequences of thylakoid-located transport proteins from *Arabidopsis* and representative cyanobacteria for each of the proteins in Table 2. Sequences from curated gene families were drawn from the following databases: ARAMEMNON, Gramene (www.gramene.org/) and Cyanobase (http://genome.microbedb.jp/cyanobase/). The thylakoid-located members were used as queries in BlastP searches [120] to NCBI and Cyanobase. BlastP searches were made to other (1) green plants, to get land plant and green alga representatives, (2) non-green plant members of Archaeplastida (includes other chloroplast-containing eukaryotes, such as glaucophytes and red algae), (3) eukaryotes and to (4) cyanobacteria, to provide context for understanding the evolution of the proteins. In some cases we also included the closest non-photosynthetic bacteria matches. By using BlastP alone, we may have failed to find sequences present only in nucleotide databases, i.e., without hypothesized or confirmed translations into amino acid sequences. Thus, our findings are necessarily limited by this caveat. For example, it is possible that the closest relatives to the green plant transport proteins have not been found in the other taxa presented in Fig. 5. Although our conclusions are subject to revision in light of new genome sequences not yet available, the existing genomes for members of critical taxa to test the hypothesis of the origin of each transport protein have been available for several years and, in some cases, for more than a decade [121–123]. Therefore, the use of BlastP alone is unlikely to affect our findings.

### Alignment

Protein sequences were initially aligned with ClustalW [124] and then inspected manually for obvious discrepancies. In some cases, the best BlastP matches from certain lineages could not be aligned with any confidence and were removed at this stage. The sequences were then trimmed to retain only the more conserved domains (the N- and C-terminal regions are often highly variable and not alignable across the sample). The sequences were realigned independently with MAFFT [125] and MUSCLE [126], in some cases with some small additional trimming, and then both alignments were subjected to phylogenetic analysis.

### Phylogenetic inference

The inference of the phylogeny was made using Bayesian analysis [127], via a Metropolis-coupled Markov chain Monte Carlo search method implemented using duplicated runs in Mr Bayes v.3.2. These analyses were run for between one and three 1–3 M generations, such that the standard deviation of split frequencies reached or dropped below 0.01. Two chains were used for each run (one cold and one heated: default settings), and a reversible model jump method was employed to explore which amino acid substitution model best fits the data. Among site rate heterogeneity was accommodated in each run by using a gamma distribution to model rate heterogeneity. Convergence among runs for each alignment was also assessed for all parameters using Tracer v.1.5. Convergence in the phylogeny was assessed among alignments, by examining the posterior probability of clades, in order to generate phylogenetic hypotheses that are robust to alignment differences. Trees drawn from the stable posterior distribution generated by Mr Bayes were summarised using Tree Annotator v.1.7.2 [http://beast.bio.ed.ac.uk/] and visualized using Fig Tree v.1.3.1 [http://tree.bio.ed.ac.uk/software/figtree/] with mid-point rooting.

## Concluding remarks

We have here reviewed the current knowledge about thylakoid channels and transporters from two model photosynthetic organisms, *Synechocystis* sp. PCC 6803 and *Arabidopsis thaliana*. The three most important TC categories in living organisms, namely channels, secondary and primary transporters, are all represented in the thylakoid membrane. There is no doubt that learning about the function and evolution of these proteins can be very important to increase our understanding of chloroplast biology and evolution, and in particular of the photosynthetic process and its regulation.

We found that, as for the envelope [113], thylakoid-located transport proteins most often have an origin in the host genome, rather than in the cyanobiont genome. Furthermore, the shifts from a non-thylakoid to a thylakoid location of these proteins has occurred at a number of different times in the history of green plant evolution. Together, these findings support the idea that the host genome has increased its control over the symbiont organelle, as has been proposed previously [113], but our findings also show that this process was not completed in a single step, nor over a short time frame. Instead, this increase of control and integration is better viewed as a process that may still be ongoing. Proteins encoded by the host genome have been recruited to serve in the thylakoid membrane repeatedly, from before the divergence of green plants from one another, to as recently as after the monocot—eudicot divergence. That thylakoid-located proteins are predominantly of host origin should not be unexpected, given the same earlier finding regarding envelope proteins [113]. The organellar genome has surrendered much of its ability to produce necessary molecules for biosynthesis, repair and photosynthetic function to the host genome. Therefore, the host genome needs to both communicate with and provide for all parts of the organelle, even as deeply as within the thylakoid lumen, separated as it is from the cytoplasm by three distinct membranes.

Some of the recruitment of proteins to serve in the thylakoid may have been a result of the increased specialization of pigment-protein composition and organization of thylakoids in green plants. However, few recruitment events can be placed in the stem green plant lineage corresponding to the common ancestor of those organisms that share this complex organization. As further transport proteins are investigated, it will be interesting to see whether the majority of these do indeed show a shift to thylakoid location at this stage of green plant evolution, or whether the increase in thylakoid complexity was of a more gradual nature, at least in terms of transport protein function.

There are many transport activities for which the responsible proteins await identification [38]. Those transport proteins localized to thylakoids by mass spectrometry-based proteomics also await validation through dedicated studies [34, 35, 37]. Cyanobacteria as well as algae are simple systems to manipulate and, therefore, ideal to use in finding some of the missing transporters. The thylakoid channels and transporters appear to play multiple roles during photosynthesis and stress response, which make them very important targets for bioenergy production and plant breeding.

### Electronic supplementary material

Below is the link to the electronic supplementary material.

Supplementary material 1 (PDF 4429 kb)

## Abbreviations

AAC: ADP/ATP carrier
CCCP: Carbonyl cyanide 3-chlorophenylhydrazone
*E. coli*: *Escherichia coli*
FCCP: Carbonylcyanide-4-trifluoromethoxyphenylhydrazone
KEA: K^+^ exchange antiporter
LHC: Light-harvesting complex
MFS: Major facilitator superfamily
NhaS: Na^+^/H^+^ antiporter
PAA: P-type ATPase
PAP: 3′-Phosphoadenosine phosphate
PAPS: 3′-Phosphoadenosine 5′-phosphosulfate
Pi: Inorganic phosphate
PHT: Phosphate transporter
PS: Photosystem
RC: Reaction center
*S. cerevisiae*: *Saccharomyces cerevisiae*
SLC17: Solute carrier 17
TAAC: Thylakoid ATP/ADP carrier
TIP: Tonoplast intrinsic protein
TPK: Tandem-pore potassium channel
∆μH^+^: Transthylakoid H^+^ gradient
