# Supplementary material for: Function and evolution of channels and transporters in photosynthetic membranes
Source: Cell Mol Life Sci. 2013 Jul 9;71(6):979–98. doi: 10.1007/s00018-013-1412-3 (PMC3928508; doi:10.1007/s00018-013-1412-3)
Supplement: Supplementary file 1 — Supplementary material 1 (PDF 4429 kb) [file 18_2013_1412_MOESM1_ESM.pdf]

Cellular and Molecular Life Sciences

Review

Function and evolution of channels and transporters in photosynthetic membranes

Bernard Pfeil*[1]*, Benoît Schoefs*[2]*, Cornelia Spetea*[1]**

[1] Department of Biological and Environmental Sciences, University of Gothenburg, 40530 Gothenburg, Sweden

[2] Mer, Molécules, Santé, LUNAM, IUML – FR FR 3473 CNRS, Université du Maine à Le Mans, Faculté des Sciences et Techniques, 72085 Le Mans cedex 9, France

* Correspondence: Cornelia Spetea. E-mail: cornelia.spetea.wiklund@bioenv.gu.se. Tel. +46-31-7869332. Fax: +46-31-7862560.

Electronic Supplementary material

Fig. S1 PAA mid-point rooted protein phylogeny inferred using Bayesian analysis.

Fig. S2 KEA mid-point rooted protein phylogeny inferred using Bayesian analysis.

Fig. S3 PHT4 mid-point rooted protein phylogeny inferred using Bayesian analysis.

Fig. S4 TPK mid-point rooted protein phylogeny inferred using Bayesian analysis.

Fig. S5 CLC mid-point rooted protein phylogeny inferred using Bayesian analysis.

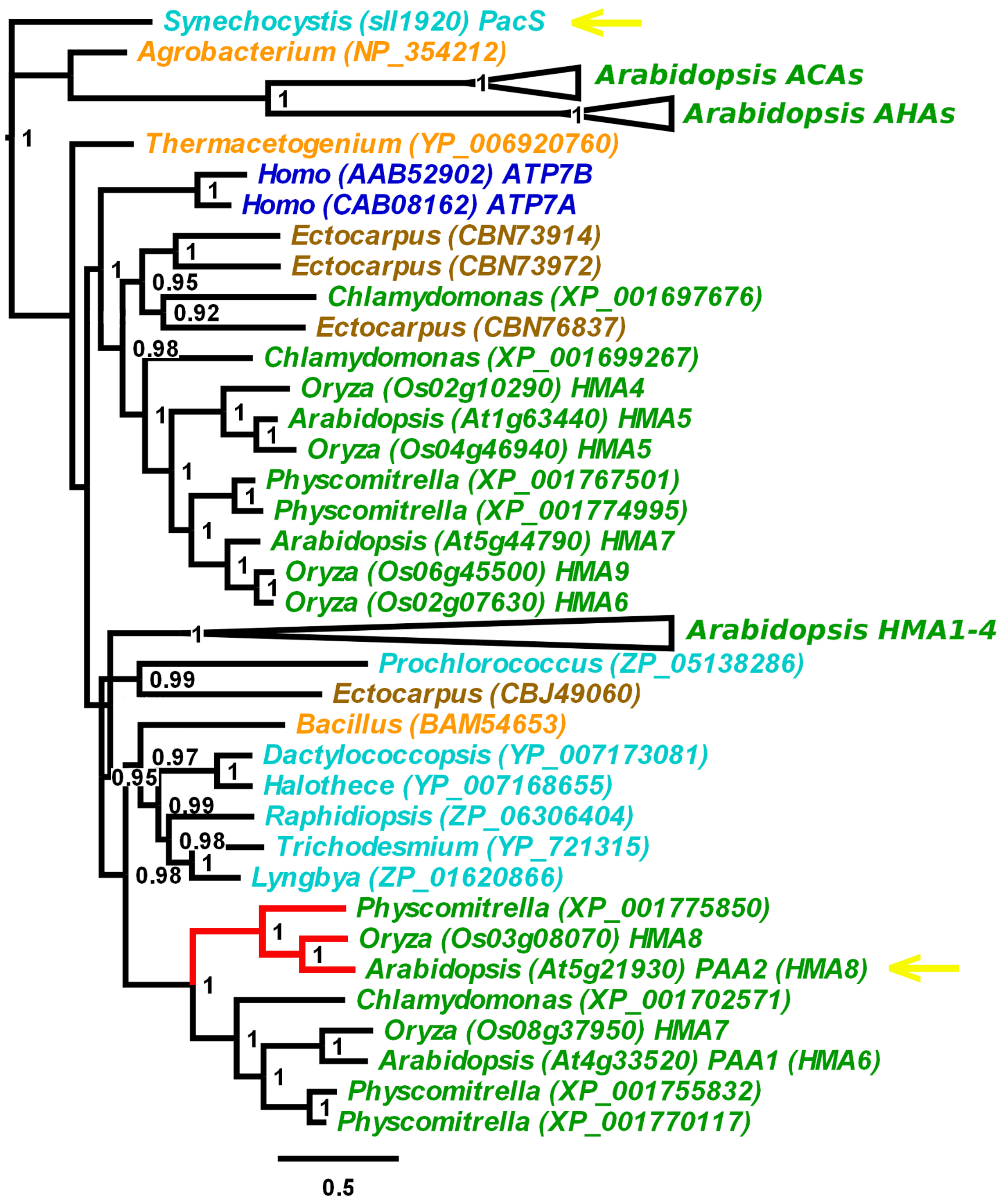

Fig. S1 PAA mid-point rooted protein phylogeny inferred using Bayesian analysis. The trees shown are maximum clade credibility trees summarised using Tree Annotator with the scale bar indicating substitutions per site. Posterior probabilities are indicated to the right of clades. Thylakoid-located proteins PAA2 and PacS are marked with yellow arrows. A group of proteins probably orthologous to *Arabidopsis* PAA2 are marked with red branches. Colours of the taxon labels indicate membership of major groups: green for green plants (green algae and land plants); red for red algae; brown for brown algae and diatoms; dark blue for opisthokonts (a large clade that includes animals and fungi); light blue for cyanobacteria; orange for other bacteria. Taxons in black do not belong to any of the named groups we discuss to test our hypothesis.

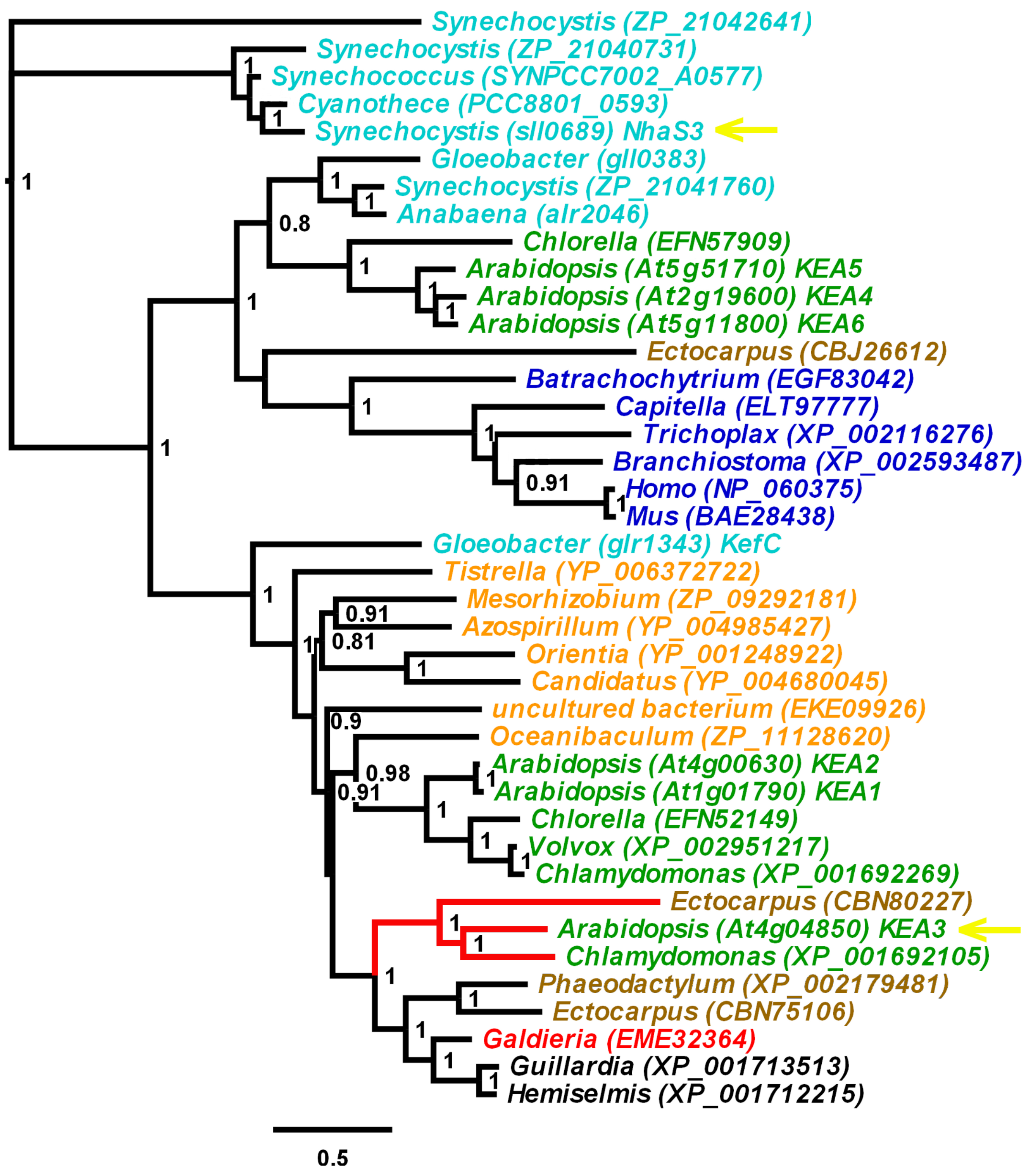

Fig. S2 KEA mid-point rooted protein phylogeny inferred using Bayesian analysis. The trees shown are maximum clade credibility trees summarised using Tree Annotator with the scale bar indicating substitutions per site. Posterior probabilities are indicated to the right of clades. Thylakoid-located proteins KEA3 and NhaS3 are marked with yellow arrows. A group of proteins probably orthologous to *Arabidopsis* KEA3 are marked with red branches. Colours of the taxon labels are as in Fig. S1.

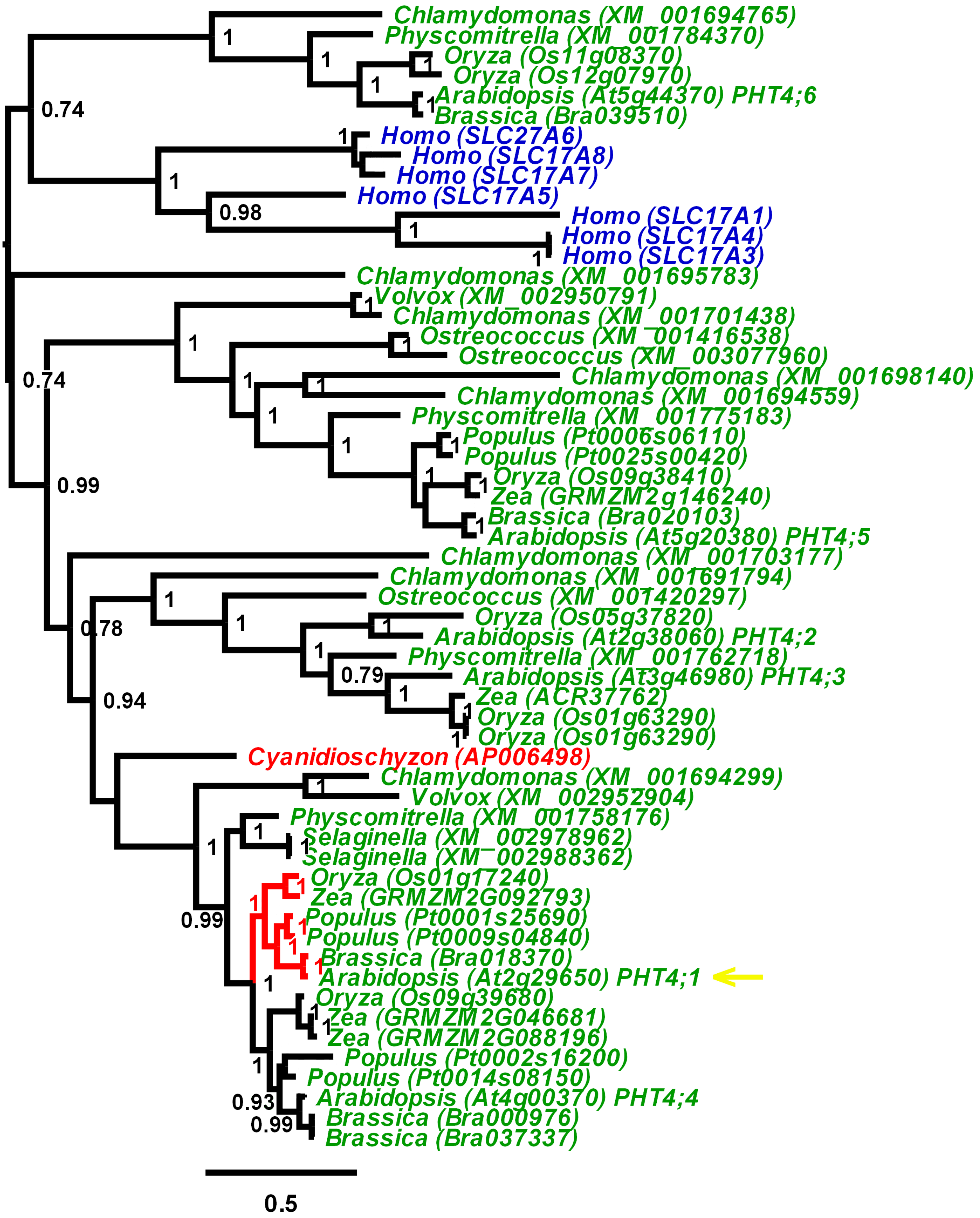

Fig. S3 PHT4 mid-point rooted protein phylogeny inferred using Bayesian analysis. The trees shown are maximum clade credibility trees summarised using Tree Annotator with the scale bar indicating substitutions per site. Posterior probabilities are indicated to the right of clades. The thylakoid-located protein PHT4-1 is marked with a yellow arrow. A group of proteins probably orthologous to *Arabidopsis* PHT4;1 are marked with red branches. Colours of the taxon labels are as in Fig. S1.

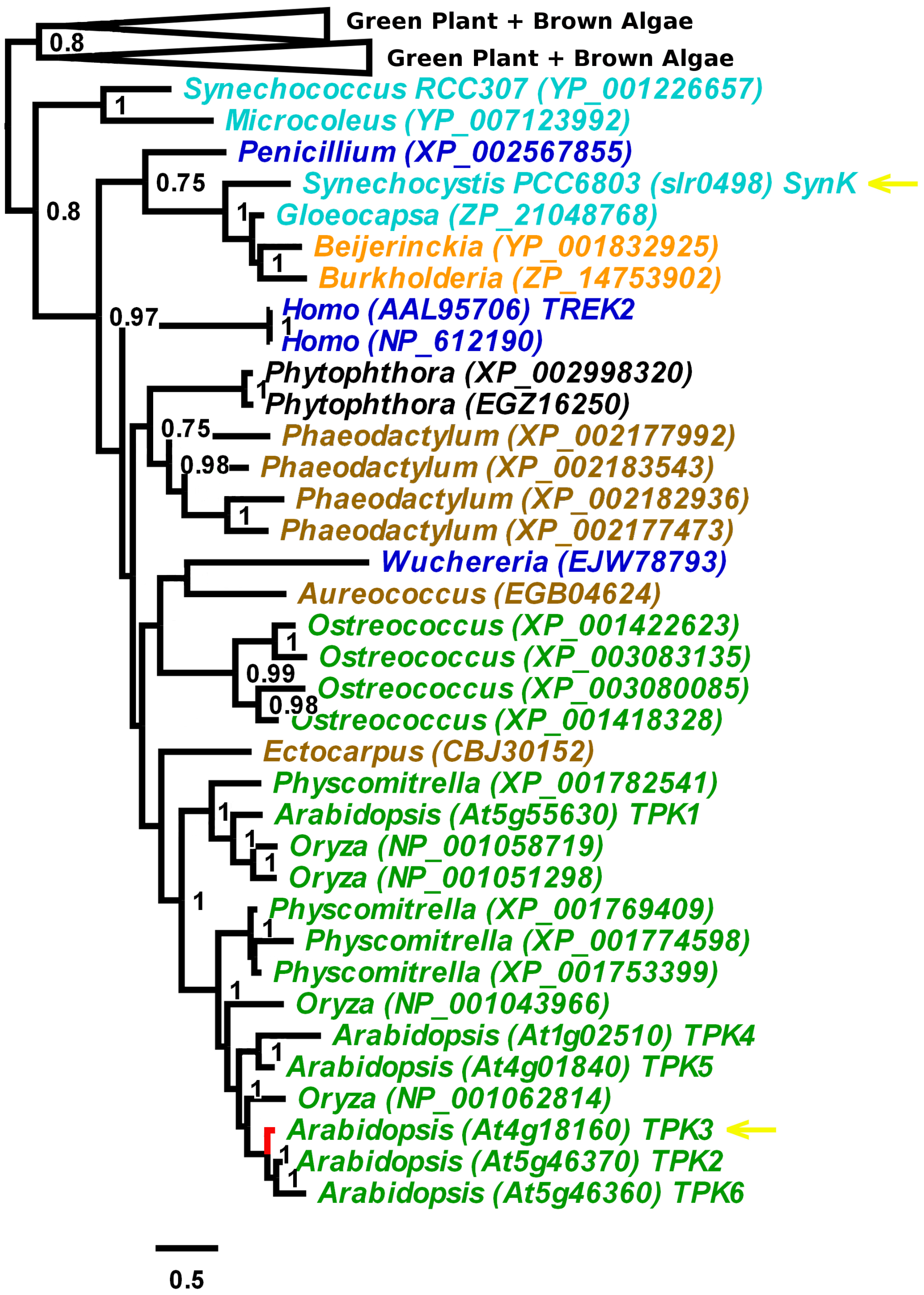

Fig. S4 TPK mid-point rooted protein phylogeny inferred using Bayesian analysis. The trees shown are maximum clade credibility trees summarised using Tree Annotator with the scale bar indicating substitutions per site. Posterior probabilities are indicated to the right of clades. Thylakoid-located proteins TPK3 and SynK are marked with yellow arrows. *Arabidopsis* TPK3, marked with a red branch, does not have any exclusive orthologues with respect to the sampling in this tree and two other *Arabidopsis* proteins (TPK2 and TPK6). Colours of the taxon labels are as in Fig. S1.

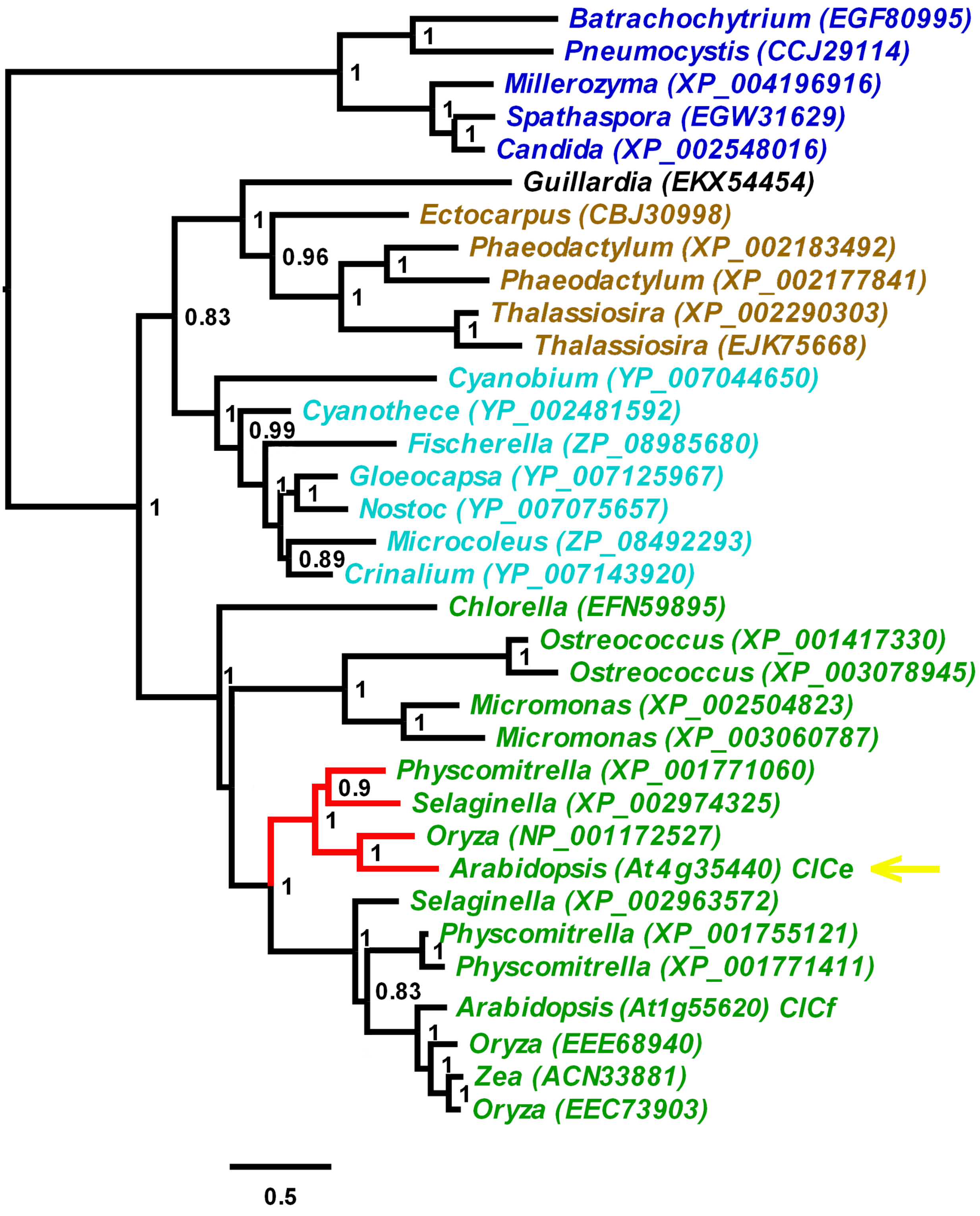

Fig. S5 CLC mid-point rooted protein phylogeny inferred using Bayesian analysis. The trees shown are maximum clade credibility trees summarised using Tree Annotator with the scale bar indicating substitutions per site. Posterior probabilities are indicated to the right of clades. The thylakoid-located protein ClCe is marked with a yellow arrow. A group of proteins probably orthologous to *Arabidopsis* ClCe are marked with red branches. Colours of the taxon labels are as in Fig. S1.
